# Time to maximum amplitude of thromboelastography can predict mortality in patients with severe COVID-19: a retrospective observational study

**DOI:** 10.3389/fmed.2024.1356283

**Published:** 2024-05-02

**Authors:** Lincui Zhong, Qingwei Lin, Longping He, Dongmei Liu, Lin Zhu, Qingbo Zeng, Jingchun Song

**Affiliations:** ^1^Intensive Care Unit, The 908th Hospital of Chinese PLA Logistic Support Force, Nanchang, Jiangxi, China; ^2^Intensive Care Unit, Huoshenshan Hospital, Wuhan, Hubei, China; ^3^Intensive Care Unit, The 940th Hospital of Chinese PLA Logistic Support Force, Lanzhou, Gansu, China; ^4^Department of Critical Care Medicine, The 944th Hospital of Chinese PLA Logistic Support Force, Jiuquan, Gansu, China

**Keywords:** thromboelastography, coronavirus, critical illness, time to maximum amplitude, mortality

## Abstract

**Objective:**

To predict mortality in severe patients with COVID-19 at admission to the intensive care unit (ICU) using thromboelastography (TEG).

**Methods:**

This retrospective, two-center, observational study involved 87 patients with PCR-and chest CT-confirmed severe COVID-19 who were admitted to at Wuhan Huoshenshan Hospital and the 908th Hospital of Chinese PLA Logistic Support Force between February 2020 and February 2023. Clinic demographics, laboratory results, and outcomes were compared between those who survived and those who died during hospitalization.

**Results:**

Thromboelastography showed that of the 87 patients, 14 were in a hypercoagulable state, 25 were in a hypocoagulable state, and 48 were normal, based on the time to maximum amplitude (TMA). Patients who died showed significantly lower α angle, but significantly longer R-time, K-time and TMA than patients who survived. Random forest selection showed that K-time, TMA, prothrombin time (PT), international normalized ratio (INR), D-dimer, C-reactive protein (CRP), aspartate aminotransferase (AST), and total bilirubin (Tbil) were significant predictors. Multivariate logistic regression identified that TMA and CRP were independently associated with mortality. TMA had a greater predictive power than CRP levels based on time-dependent AUCs. Patients with TMA ≥ 26.4 min were at significantly higher risk of mortality (hazard ratio 3.99, 95% Confidence Interval, 1.92–8.27, *p* < 0.01).

**Conclusion:**

TMA ≥26.4 min at admission to ICU may be an independent predictor of in-hospital mortality for patients with severe COVID-19.

## Introduction

The world continues to experience outbreaks of novel coronavirus disease-2019 (COVID-19) (1), for which the overall case-fatality rate may be around 2.3%, increasing to 49–62% among critical cases (2, 3). One of the major causes of death among COVID-19 patients admitted to the intensive care unit is severe coagulation dysfunction (4). Patients who die in hospital tend to show higher levels of D-dimer and fibrin degradation product (FDP) on admission than those who survive hospitalization (5). In fact, a high incidence of arterial and venous thrombotic complications in COVID-19 patients including pulmonary embolism (PE), deep-vein thrombosis (DVT), ischemic stroke, myocardial infarction, or systemic arterial embolism gave rise to increased mortality (6–9). Most seriously, more than 70% of severe COVID-19 patients who developed disseminated intravascular coagulation (DIC) died in hospital (10).

While routine laboratory assays of plasma can provide valuable information about the hemostasis of patients with COVID-19, they have significant limitations in quantifying hypercoagulable states and evaluating cellular contribution to hemostasis by platelets and red blood cells (RBC) (11, 12). TEG may provide more comprehensive analysis of hemostatic capacity, because it can assess the coagulation function of cells and individual proteins (13, 14). Two coagulation states of sepsis-induced DIC were identified by TEG tracings: a hypercoagulable state of early DIC and a hypocoagulable state of late DIC, which provided evidence for further anticoagulation or alternative therapy (15, 16). Unfortunately, prognostic implications of TEG parameters in COVID-19 patients have not been reported. We compared the findings between patients who survived or died during hospitalization, and we assessed whether TEG parameters may help identify patients at higher risk of mortality in the ICUs.

## Methods

### Participants

This study retrospectively analyzed 87 patients with severe COVID-19 who were admitted to the ICU of Wuhan Huoshenshan Hospital (Wuhan, China) and the 908th Hospital of Chinese PLA Logistic Support (Nanchang, China) between February 2020 and February 2023. All 87 patients were diagnosed with COVID-19 based on World Health Organization interim guidance (17) and detection of SARS-CoV-2 RNA in the hospital’s clinical laboratory. In addition, all patients were defined as having severe disease because they were admitted to the ICU and required mechanical ventilation or they experienced shock or organ failure (18). We excluded patients with congenital coagulation disorders, or pre-existing liver failure, as well as those who were taking systemic anticoagulation or antiplatelet medications.

### Data collection

Data were collected on age, sex, in-hospital survival, and the following laboratory results on admission: complete blood count, analyzed using an XE-2100 Hematology Analyzer (Sysmex, Japan); blood chemistry, analyzed on an AU5800 system (Beckman Coulter, United States); coagulation status, analyzed using the CS5100 system (Sysmex, Japan); and thromboelastography after kaolin-activation, performed using an LEPU-8800 system (LEPU, China).

Patients were analyzed by TEG, and the following indices were determined (19–21): reaction time (R time), defined as the interval from when the blood was placed in the analyzer until the start of fibrin formation; kinetic time (K time), defined as the time to reach a certain level of clot strength; α angle, which reflects the level of fibrinogen; maximum amplitude (MA), which reflects platelet function; clot lysis 30 min following maximal amplitude (LY30), which reflects clot lysis; TMA, defined as the time needed to form a stable clot. Patients were classified as being in a hypercoagulable, normal, or hypocoagulable state based on whether TMA was, respectively, < 23, 23–33, or > 33 min (22, 23).

### Statistical analysis

Data were reported as mean ± standard deviation, median (interquartile range), or *n* (%), as appropriate. Data were analyzed using R 4.2.1 (R Core Team, Vienna, Austria), and differences associated with *p* < 0.05 were considered significant. Continuous data were compared between survivors and non-survivors using Student’s *t* test if data were normally distributed, or using the Mann–Whitney U test if the data were skewed. Categorical data were compared using the chi-squared test. Random Forest and correlation matrix were applied to select features. The ability of selected features to predict mortality was assessed using logistic regression. The ability of TMA and CRP to predict mortality in our sample was further assessed by the area assessed by the time-dependent receiver operator characteristic curve (AUC). Survival of patients below or above the optimal TMA cut-off value was compared using Kaplan–Meier curves and the log-rank test. Risk of mortality was expressed in terms of a hazard ratio (HR) and corresponding 95% confidence interval (95% CI).

## Results

Of the 104 patients initially assessed for enrollment, one hemophilia B patient, three patients with cirrhosis, five patients receiving rivaroxaban and eight patients receiving aspirin were excluded. Finally, the final analysis included 87 patients with severe COVID-19, who were 73 (63, 89) years old and were predominantly male (*n* = 55). By the end of February, 2023, 56 (64.4%) patients had been discharged and 31 (35.6%) patients had died.

While patients who died showed similar age and gender as those who survived, those who died showed significantly greater inflammation on admission, based on counts of white blood cells count and neutrophils as well as the level of C-reactive protein (Table 1). Based on aspartate aminotransferase and serum creatinine levels, liver and kidney function was significantly worse on admission to non-survivors compared with survivors (Table 1). They also showed significantly lower platelet count, longer activated partial thromboplastin time and prothrombin time, and higher D-dimer levels, indicating worse coagulation function compared to survivors on admission.

**Table 1:** Characteristics of severe COVID-19 patients at admission.

Variable	Normal range	All (*n* = 87)	Survivors (*n* = 56)	Non-survivors (*n* = 31)	*p*
Age (years)		73 (63, 89)	73.5 (65, 89)	72 (60, 87.5)	0.598
Male/female		55/32	36/20	19/12	0.964
White blood cells (×10^9^/L)	3.5–9.5	8.6 (6.4, 13.6)	7.7 (6.2, 12.5)	10.3 (7.9, 16.1)	0.03
Neutrophils (×10^9^/L)	2.5–7.5	7.8 (5.1, 11.4)	6.3 (4.9, 10.8)	9.7 (6.8, 14.9)	0.045
Lymphocytes (×10^9^/L)	0.8–4	0.8 (0.5, 1.1)	0.8 (0.5, 1.1)	0.6 (0.4, 1.1)	0.373
Hemoglobin (g/L)	115–150	93.6 ± 21.2	93 ± 22.8	94.6 ± 18.2	0.72
CRP (mg/L)	0–4	65.4 (26.6, 103.3)	55.2 (17.6, 81.4)	103.3 (44.1, 144.4)	< 0.001
TBil (μmoI/L)	0–21	12.0 (7.4–23.4)	11.2 (7.0–17.5)	13.5 (10.7–45.7)	0.072
AST (U/L)	7–45	32.4 (23.2, 84.3)	29 (22.6, 47.4)	63.9 (28.9, 170.1)	0.006
ALT (U/L)	7–40	27.8 (17.7, 50.9)	23.6 (17.2, 40.4)	37.9 (20.4, 93)	0.039
SCr (μmoI/L)	41–81	75.4 (53.8, 156.1)	67.1 (48.7, 104.8)	100.8 (70.2, 179.3)	0.02
PLT (×10^9^/L)	125–350	172 (72.5, 235.5)	195.5 (105, 247.8)	120 (64, 194)	0.048
APTT (s)	21–37	32.7 (29.6, 38.3)	31.7 (28.7, 35.7)	37.2 (31.5, 42.9)	0.002
PT (s)	9.2–15	14.2 (12.8, 15.9)	13.9 (12.7, 15)	15.3 (13.6, 20)	0.002
INR	0.8–1.25	1.2 (1.1, 1.3)	1.1 (1.1, 1.2)	1.3 (1.1, 1.7)	0.002
FIB (g/L)	2–4	4.9 (3.8, 13.5)	4.8 (3.8, 8.5)	5.3 (4.1, 13.9)	0.28
TT (s)	10–20	15.1 (4.6, 16.3)	14.9 (10.8, 16)	15.2 (4.1, 17.5)	0.556
D-Dimer (μg/mL)	0–0.55	2.3 (0.8, 5)	1 (0.4, 3)	5.3 (2.7, 16)	<0.001

Values are *n*, mean ± SD, or median (interquartile range), unless otherwise noted. ALT, Alanine aminotransferase; APTT, Activated partial thromboplastin time; AST, Aspartate aminotransferase; CRP, C-reactive protein; FIB, Fibrinogen; HGB, Hemoglobin concentration; INR, International normalized ratio; PLT, Platelet count; PT, Prothrombin time; SCr, Serum creatinine; TBil, Total bilirubin; TT, Thrombin time.

**Figure 1:**
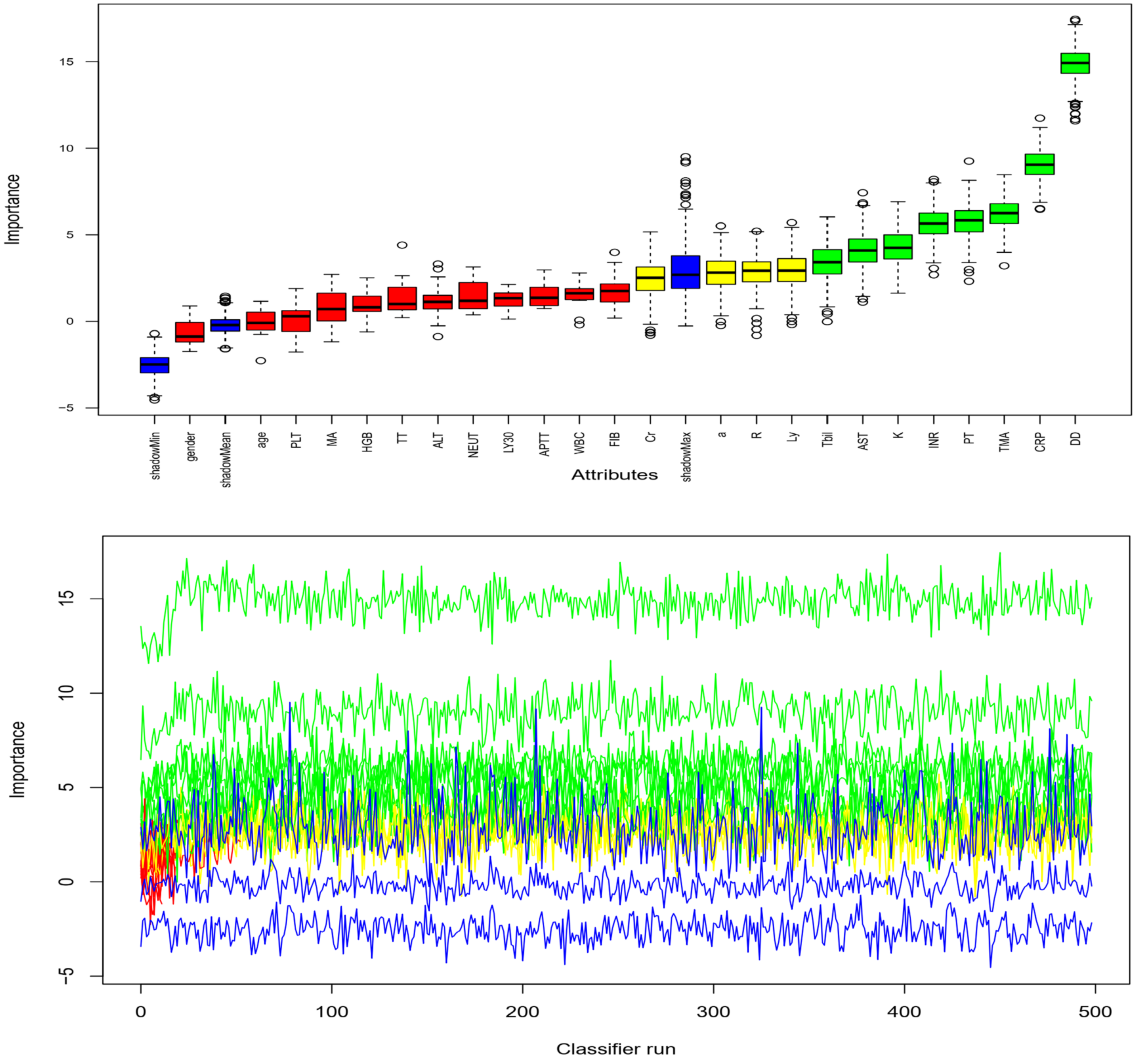
Machine learning analysis and selection of biomarker for prognosis.

**Figure 2:**
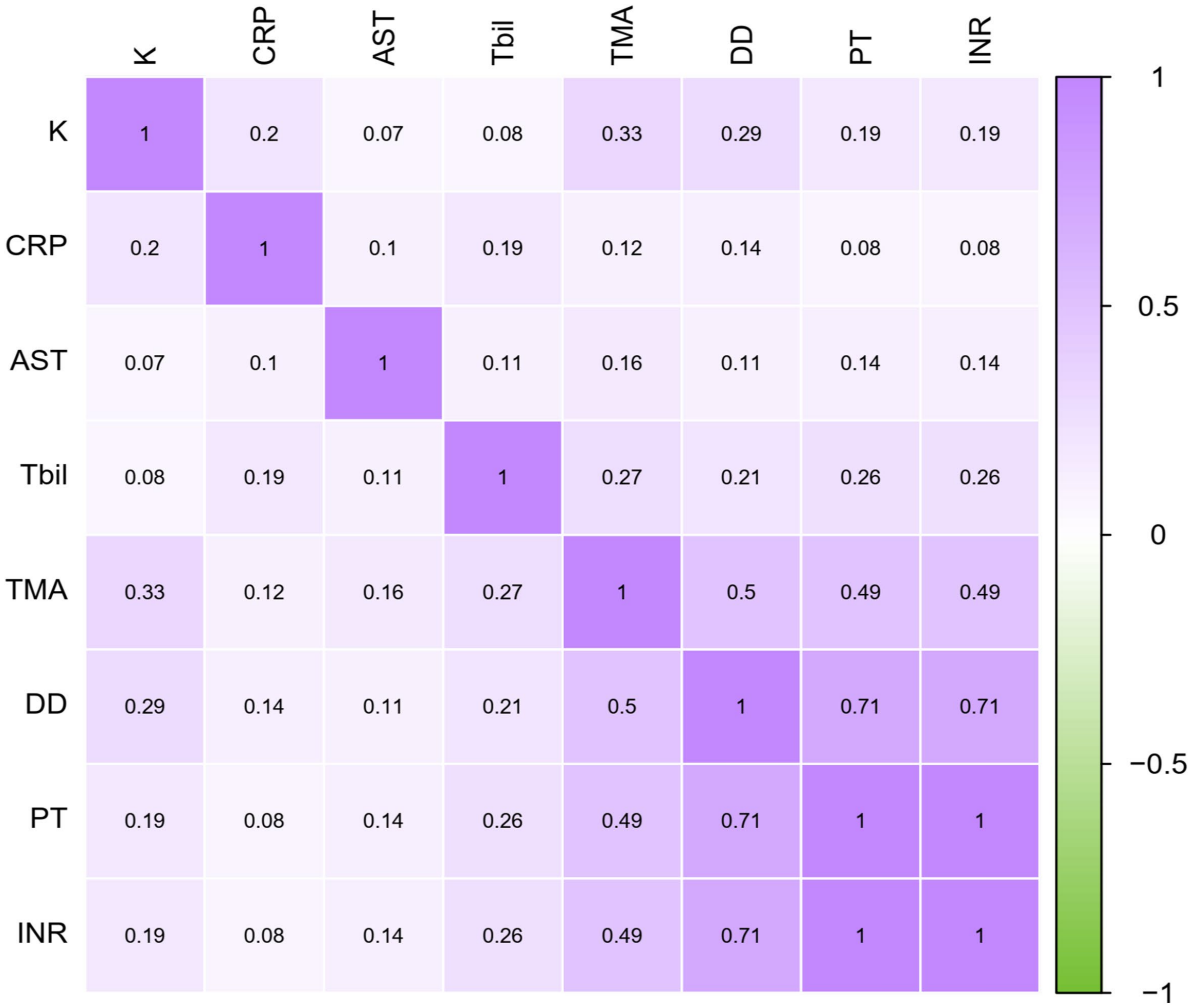
Correlation matrix.

**Figure 3:**
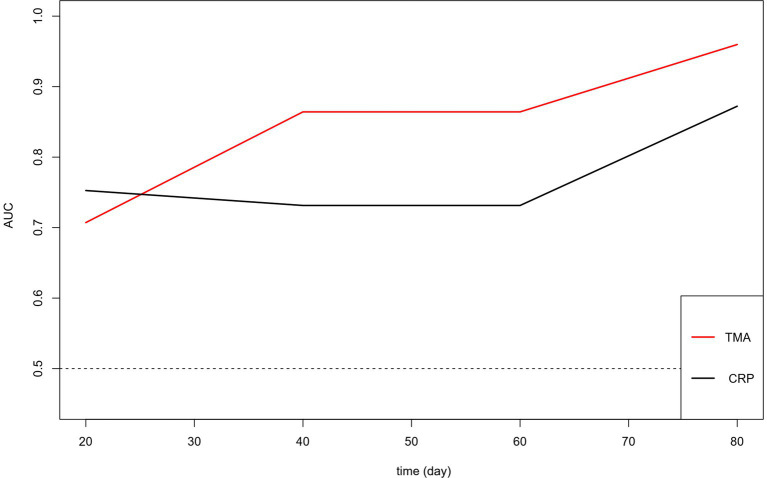
Time-dependent ROC for TMA and CRP for predicting mortality. AUC, Area under the curve; ROC, Receiver operating curves.

**Figure 4:**
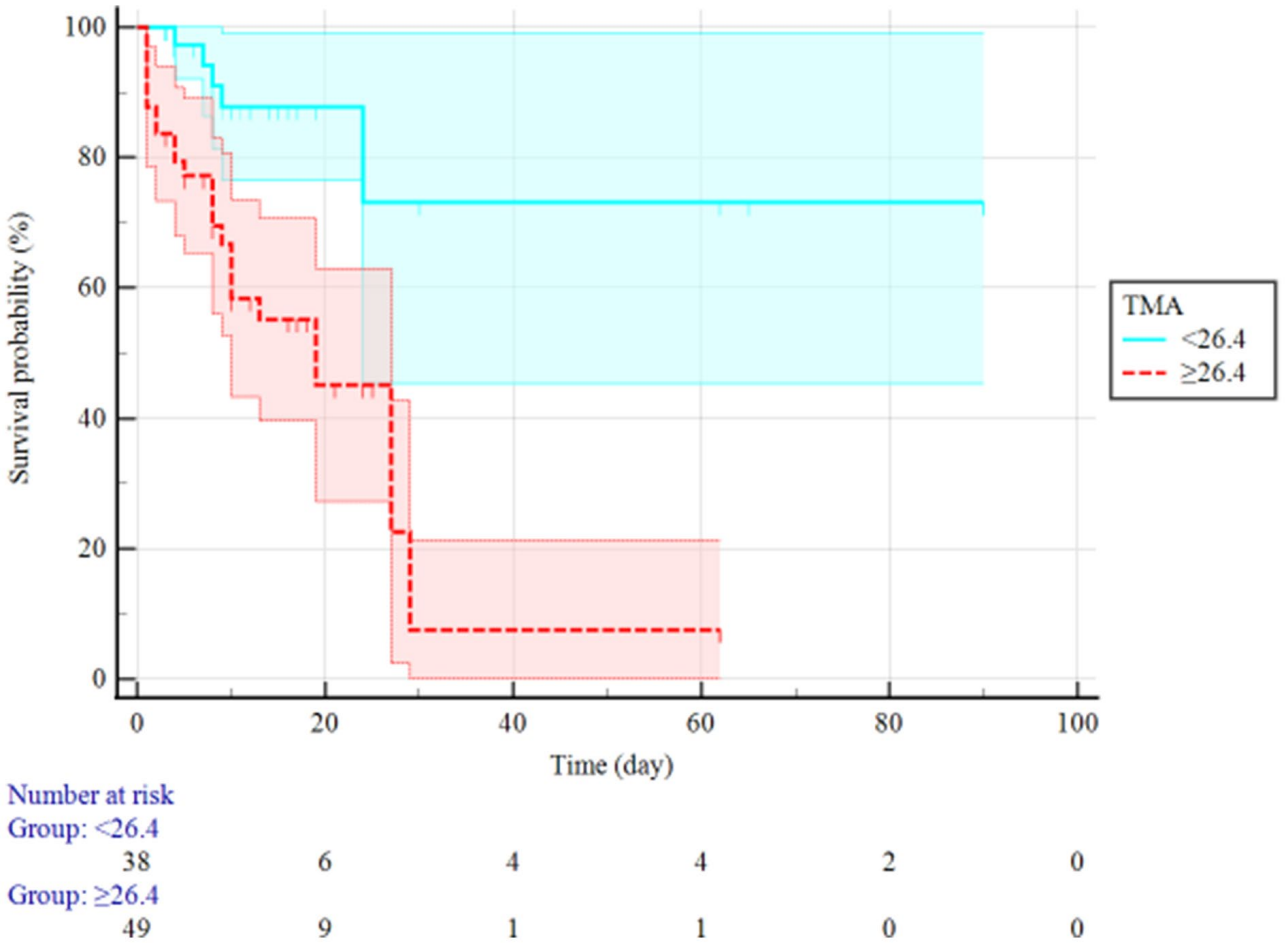
Kaplan–Meier survival curve of resected in severe COVID-19 patients with long TMA (≥26.4 min) and short TMA (<26.4 min).

Based on thromboelastography, 14 patients were in a hypercoagulable state and 25 were in a hypocoagulable state on admission. Patients who died during the study period showed lower α angle, as well as longer R-time, K-time, MA, and TMA than those who survived (Table 2). The random forest is an important feature selection method, which is frequently applied to select key variables or features. In this study, there were 24 clinical variables which were selected for further analysis. Random forest selection showed eight variables were significant predictors: K, TMA, PT, INR, DD, CRP, AST, and Tbil (Figure 1). The indices were used for correlation matrix analysis, which showed high correlation coefficients for PT, INR, and DD (Figure 2). PT, INR, and DD were not included in the logistic regression analysis because of multicollinearity problems. Tbil was considered from a clinical specialty perspective and was also not included in the logistic regression analysis. The remaining four significant variables were directly analyzed by multivariate logistic regression, and the results identified that TMA and CRP were independently associated with mortality (Table 3). Figure 3 showed AUCs for TMA and CRP using time-dependent ROC analysis. TMA had greater predictive power than the CRP for in-hospital mortality based on time-dependent AUCs. Survival analysis was applied to investigate the impact of TMA on mortality. Stratifying patients using this cut-off showed that those with TMA ≥ 26.4 min were the significant predictor of subsequent deaths (Log-rank = 13.84, *p* < 0.001, HR = 3.99, 95%CI 1.92–8.27). Notably, TMA was significantly related to in-hospital mortality and the median survival time reached 27 days (Figure 4).

**Table 2:** Thromboelastography parameters of severe COVID-19 patients at admission.

Parameter	Normal range	All (*n* = 87)	Survivors (*n* = 56)	Non-survivors (*n* = 31)	*p*
R-time (min)	5–10	7.2 (5.8, 10.5)	6.8 (5.5, 8.7)	9.4 (6.4, 12.3)	0.014
K-time (min)	1–3	1.8 (1.3, 4)	1.6 (1.2, 2.3)	3.1 (1.8, 5.2)	< 0.001
α angle (°)	53–72	60.5 (42.3, 69.8)	63.9 (53.4, 71.2)	50.3 (38.7, 66.2)	0.011
MA (mm)	50–70	65.2 (53.7, 71.8)	68.5 (58.4, 72.9)	62.9 (42, 68.8)	0.017
LY30 (%)	0–8	0 (0, 0.3)	0.1 (0, 0.5)	0 (0, 0.1)	0.056
TMA (min)	23–33	27.8 (24.8, 35)	25.9 (24, 30)	34.3 (27.3, 38.8)	< 0.001

Values are *n*, mean ± SD, or median (interquartile range), unless otherwise noted. K-time, Kinetic-time; LY30, Clot lysis 30 min following thromboelastography maximal amplitude; MA, Maximum amplitude; R-time, Reaction time; TMA, Time to maximum clot strength.

**Table 3:** Multivariate logistic regression to identify parameters associated with mortality in severe COVID-19 patients.

Parameter	Odds ratio (95%CI)	*p*
TMA (min)	1.068 (1.016–1.122)	0.01
AST (U/L)	1.002 (0.999–1.006)	0.22
CRP (mg/L)	1.011 (1.003–1.018)	0.006
K (min)	1.053 (0.956–1.159)	0.295

## Discussion

To our knowledge, this is the first assessment of whether thromboelastographic parameters can be used to identify patients with severe COVID-19 who are more likely to die within 30 days after ICU admission. Our results suggest that TMA may be useful as a prognostic indicator, which may reflect its ability to assess interaction between coagulation factors and platelets (24). Indeed, we found that TMA correlated with prothrombin time, international normalized ratio, and D-Dimer. Whatever the explanation, our data suggest that using TMA to screen COVID-19 patients for mortality risk may help optimize limited healthcare resources and provide timelier patient management and treatment, ultimately leading to better outcomes.

Studies have linked COVID-19 to hypercoagulability and decreased fibrinolysis, yet we found that only 14 of our 87 patients with severe disease were in a hypercoagulable state, which is similar to the proportion reported for a sample of patients from Asian (25), but much lower than the 50% reported for patients from Caucasian (26). Conversely, a high proportion of our non-survivors were in a hypocoagulable state which manifested as reduced platelet count, longer activated partial thromboplastin time and prothrombin time, and elevated D-dimer levels. Thromboelastography confirmed this hypocoagulable state by indicating reduced α angle as well as longer R-time, K-time, and TMA. Similarly, our incidence of hypocoagulable state is significantly higher than that in the COVID-19 patients from Caucasian (26). Ethnicity may help explain these differences, since Caucasians and African-Americans are generally at higher risk of thromboses than Chinese (13). Whatever the explanation, our results suggest that hypocoagulability is associated with mortality among patients with severe COVID-19, consistent with a previous report linking hypocoagulability to COVID-19 severity (14).

Many patients with severe pneumonia suffer disseminated intravascular coagulation and multiple organ failure when vascular endothelium, platelets, and leukocytes become activated and dysregulate the production of thrombin systemically and in the lungs (27). A similar process may occur in COVID-19, which has also been linked to disseminated intravascular coagulation and multiple organ failure (28, 29). Thus, treatments to restore or regulate hemostasis may benefit individuals infected with coronavirus (30). For example, administering heparin to patients with severe COVID-19 with extremely high D-dimer levels can decrease mortality (31). While elevated D-dimer levels can signal the activation of coagulation and fibrinolysis (32), they can also occur due to bleeding. Therefore, thromboelastography may be better for determining the timing of anticoagulation therapy (33).

Our findings should be interpreted with caution in light of several limitations. First, our sample was retrospective and came from two centers. Second, we did not perform thromboelastography multiple times during the disease course. Third, we did not provide the number of days between the onset of symptoms and hospitalization for severe patients, due to the difficulty in obtaining their medical history upon admission. Our results should be verified and extended in larger studies with longitudinal monitoring.

Despite these limitations, our study demonstrates the usefulness of thromboelastography for assessing the coagulation state of patients with severe COVID-19. It also provides the first evidence that the thromboelastographic parameter TMA may accurately identify severe COVID-19 patients at higher risk of dying.

## Data availability statement

The original contributions presented in the study are included in the article/Supplementary material, further inquiries can be directed to the corresponding author/s.

## Ethics statement

The studies involving humans were approved by the Ethics Committee of Wuhan Huoshenshan Hospital and the 908th Hospital of Chinese PLA Logistic Support Force. The studies were conducted in accordance with the local legislation and institutional requirements. The ethics committee/institutional review board waived the requirement of written informed consent for participation from the participants or the participants’ legal guardians/next of kin because all patients or their legal guardians gave written consent, at the time of admission, for patients’ anonymized data to be analyzed and published for research purposes.

## Author contributions

LZho: Writing – original draft. QL: Data curation, Formal Analysis, Writing – original draft. LH: Software, Writing – original draft. DL: Data curation, Writing – review & editing. LZhu: Data curation, Writing – review & editing. QZ: Validation, Writing – review & editing. JS: Conceptualization, Funding acquisition, Writing – review & editing.
